# The intake of solid fat and cheese may be associated with a reduced risk of Helicobacter pylori infection status: a cross-sectional study based on NHANES 1999–2000

**DOI:** 10.1186/s12879-024-09392-z

**Published:** 2024-05-14

**Authors:** Huan Zhang, Chao Xu, Ju Zhang, Jumei Yin, Nuo Yao, Qimeng Pang, Zhihua Liu, Chenchen Wang, Yongquan Shi, Lei Shang, Zheyi Han

**Affiliations:** 1 Department of Gastroenterology, Air Force Medical Center, Fourth Military Medical University, Beijing, China; 2 State Key Laboratory of Holistic Integrative Management of Gastrointestinal Cancers and National Clinical Research Center for Digestive Diseases, Xijing Hospital of Digestive Diseases, Fourth Military Medical University, Xi’ an, 710032 China; 3 Department of Health Statistics, School of Preventive Medicine, Fourth Military Medical University, Xi’an, China; 4 Department of Knee Joint Surgery, Honghui Hospital, Xi’an Jiaotong University, Xi’an, China; 5 Postgraduate Department, Xi’an Medical University, Xi’an, Shaanxi 710021 China; 6 Department of Critical Care Medicine, the 940th Hospital of Joint Logistics Support Force of PLA, Lanzhou, Gansu 730050 China

**Keywords:** Dietary pattern, Helicobacter pylori, NHANES

## Abstract

**Background:**

Diet plays an important role in *Helicobacter pylori* (HP) infection, and our objective was to investigate potential connections between dietary patterns, specific food groups, and HP infection status in U.S. adults.

**Methods:**

The data for this study was obtained from the NHANES (National Health and Nutrition Survey) database for the year 1999–2000. This cross-sectional study involved the selection of adults aged 20 years and older who had undergone dietary surveys and HP testing. Factor analysis was employed to identify dietary patterns, and logistic regression models were utilized to assess the association between these dietary patterns and specific food groups with HP infection status.

**Result:**

Based on the inclusion and exclusion criteria, our final analysis included 2,952 individuals. The median age of participants was 51.0 years, and 48.7% were male. In the study population, the overall prevalence of HP infection was 44.9%. Factor analysis revealed three distinct dietary patterns: High-fat and high-sugar pattern (including solid fats, refined grains, cheese, and added sugars); Vegetarian pattern (comprising fruits, juices, and whole grains); Healthy pattern (encompassing vegetables, nuts and seeds, and oils). Adjusted results showed that the high-fat and high-sugar pattern (OR = 0.689, 95% CI: 0.688–0.690), vegetarian pattern (OR = 0.802, 95% CI: 0.801–0.803), and healthy pattern (OR = 0.717, 95% CI: 0.716–0.718) were all linked to a lower likelihood of HP infection. Further analysis of the high-fat and high-sugar pattern revealed that solid fats (OR = 0.717, 95% CI: 0.716–0.718) and cheese (OR = 0.863, 95% CI: 0.862–0.864) were protective factors against HP infection, while refined grains (OR = 1.045, 95% CI: 1.044–1.046) and added sugars (OR = 1.014, 95% CI: 1.013–1.015) were identified as risk factors for HP infection.

**Conclusion:**

Both the Vegetarian pattern and the Healthy pattern are associated with a reduced risk of HP infection. Interestingly, the High-fat and High-sugar pattern, which is initially considered a risk factor for HP infection when the score is low, becomes a protective factor as the intake increases. Within this pattern, animal foods like solid fats and cheese play a protective role, while the consumption of refined grains and added sugars increases the likelihood of HP infection.

**Supplementary Information:**

The online version contains supplementary material available at 10.1186/s12879-024-09392-z.

## Introduction

*Helicobacter pylori* (HP) has been a subject of significant concern since its discovery by Australian scholars Marshall and Warren in 1983 [1]. It affects more than half of the world’s population. Epidemiological studies indicate that the global HP infection rate is approximately 50%, and in developing countries, the rate is even higher, exceeding 70% [2, 3].HP infection is defined as an infectious disease according to the International HP Kyoto Consensus (2015) [4] and Masstricht VI Consensus (2022) [5]. It is recognized by the World Health Organization (WHO) and the International Agency for Research on Cancer (IARC) and is classified as a Group 1 carcinogen [6]. It is associated with various gastric conditions, such as peptic ulcer disease, gastric adenocarcinoma, and gastric mucosa-associated lymphoid tissue lymphoma, as well as extra-gastric diseases like metabolic syndrome, diabetes, and non-alcoholic liver disease [7].

Since the 1990s, researchers have increasingly focused on the connection between diet and HP. As research has progressed, numerous foods and ingredients have been linked to HP infection, including milk [8], honey [9], tea [9], garlic [10], soft drinks [11], and more. However, people’s daily diets are highly intricate, and the foods and nutrients they consume inevitably interact with one another. Therefore, dietary indices and dietary patterns are more suitable for understanding the relationship between HP and diet than evaluating individual foods or ingredients. Two separate studies in Iran have shown that the dietary antioxidant index [12] and glycemic index [13] are associated with HP infection. Additionally, Shi et al. [14] demonstrated that increased levels of the dietary inflammatory index are linked to a higher risk of HP infection in an American adult population. Although dietary indices can be complex to grasp and may be influenced by prior knowledge, dietary patterns are relatively easier to interpret.

Two observational studies in China have found that certain dietary patterns, such as a grain-vegetable pattern rich in whole grains and vegetables or a protein/cholesterol pattern characterized by a high intake of animal offal, animal blood, fish, seafood, and poultry, are associated with a reduced prevalence of *Helicobacter pylori* infection [15, 16]. Currently, the only two studies on the association between dietary patterns and HP have been conducted in China, and there are substantial differences between Chinese and Western diets. Since dietary patterns are influenced by geography and culture, it is essential to investigate the relationship between dietary patterns and HP infection in American adults in order to provide more accurate dietary recommendations for HP prevention and treatment.

## Methods

### Data collection and research population

**Fig. 1:**
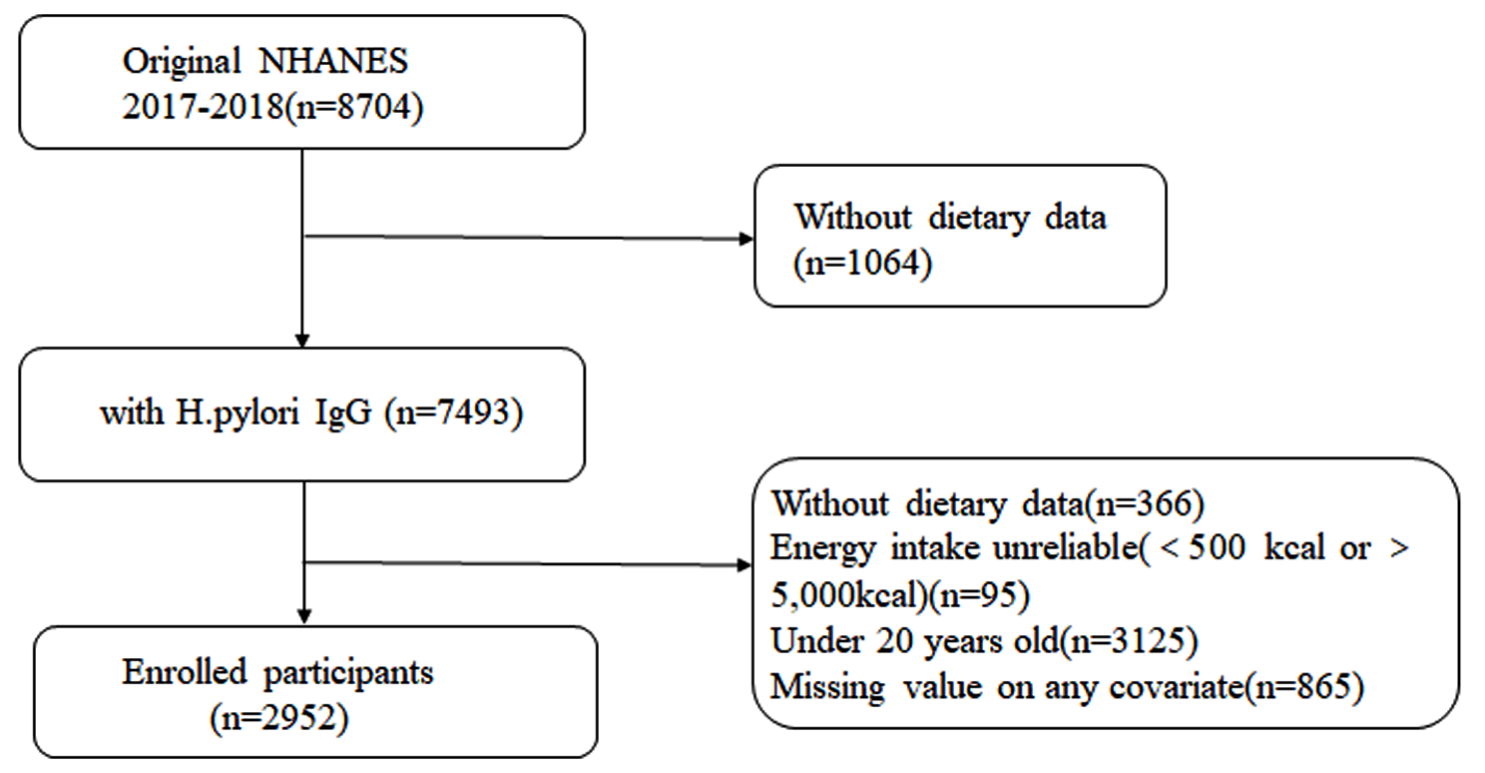
Flow chart for inclusion and exclusion of the study population

The data we analyzed was sourced from the National Health and Nutrition Examination Survey (NHANES) conducted in 1999–2000. NHANES is an ongoing survey program developed and administered by the National Center for Health Statistics (NCHS), which is part of the Centers for Disease Control and Prevention (CDC). In the 1999–2000 survey cycle, a total of 9,965 participants were excluded from our analysis. The reasons for exclusion included a lack of *Helicobacter pylori* antibody data (*n* = 2,472), absence of dietary data (*n* = 366), age less than 20 years old (*n* = 3,125), unreliable energy intake (less than 500 kcal or more than 5,000 kcal) (*n* = 95) [17], and missing values (*n* = 865) for any covariates in the model. Ultimately, 2,952 eligible subjects were included in our study. (Fig. 1)

### Dietary pattern

NHANES employs a 24-hour dietary recall method to gather information about the types and quantities of food and beverages consumed by Americans. Subsequently, the Food Pattern Equivalent Database (FPED) is used to convert this dietary data into equivalent quantities for 32 food groups [18]. As FPED provides data in terms of food and beverage ingredients per 100 g, individual food intake quantities are adjusted by dividing them by 100 g and then multiplying them by the corresponding equivalent quantity in FPED. To streamline the analysis, we excluded five groups that represented the sum of various food subgroups (for instance, “Total Red and Orange Vegetables” included subgroups like “Tomatoes” and “Other Red and Orange Vegetables”). In the end, we worked with a total of 27 distinct food groups: Fruit, Fruit Juice, Dark Green Vegetables, Tomatoes, Other Red and Orange Vegetables, Potatoes, Other Starchy Vegetables, Other Vegetables, Legumes, Whole Grains, Refined Grains, Meat, Cured Meat, Organ Meat, Poultry, Seafood High in n-3 Fatty Acids, Seafood Low in n-3 Fatty Acids, Eggs, Soy Products, Nuts and Seeds, Milk, Yogurt, Cheese, Oils, Solid Fats, Added Sugars and Alcoholic Drinks. To identify dietary patterns, exploratory factor analysis was applied to these 27 food groups.

Review the scree plot and choose factors with eigenvalues exceeding 1. Apply Varimax rotation to facilitate the interpretation of identified patterns and reduce correlations between factors. Analyze the factor loadings to identify the primary contributors to each dietary pattern. Calculate factor scores for individuals based on factor loadings and their actual food intake. Finally, name the derived dietary pattern based on the food groups with the highest factor loadings (loadings>0.2) on that specific factor.

### Evaluation of HP infection status

The Wampole Laboratory’s (Wampole) HP IgG enzyme-linked immunosorbent assay (ELISA) is designed to detect and quantitatively determine the concentration of HP IgG antibodies in human serum [14]. In comparison to other antibody serological tests, such as immunofluorescence, complement binding, hemagglutination, and radioimmunoassay, ELISA exhibits similar sensitivity, specificity, and repeatability. A serum IgG antibody value below 0.9 is considered negative, while an IgG value equal to or greater than 0.9 is considered positive, consistent with previous studies [19].

### Other covariates

Our study examined various factors that could potentially impact HP infection, including family demographic information, lifestyle habits, and self-reported health status. Family demographic information included gender (male/female), age (in years), race (Mexican American, Other Hispanic, Non-Hispanic white, Non-Hispanic Black, Other Race), education level (Less Than 9th Grade, 9-11st Grade, High School Grade/Equivalent, Some College or Associate’s Degree, College Degree or above), and poverty income ratio (PIR). Drinking status was categorized as follows: Never (consumed fewer than 12 drinks in a lifetime), Former (consumed at least 12 drinks in one year but didn’t drink in the last year or consumed at least 12 drinks in a lifetime), Mild (consumed at least 1 drink per day for females or at least 2 drinks per day for males), Moderate (consumed at least 2 drinks per day for females or at least 3 drinks per day for males, or engaged in binge drinking on at least 2 days per month) and Heavy (consumed at least 3 drinks per day for females or at least 4 drinks per day for males) [20]. Smoking status was classified as: Never (smoked fewer than 100 cigarettes in a lifetime), Former (smoked more than 100 cigarettes in a lifetime but currently do not smoke at all), Current (smoked more than 100 cigarettes in a lifetime and currently smoke some days or every day) [20]. This information helped us assess potential associations between these factors and HP infection. We also considered Body Mass Index (BMI, Kg/m^2^) in our analysis. Current medical conditions taken into account included diabetes (yes/impaired fasting glucose (IFG)/no), hypertension (yes/no), heart disease (yes/no), and stroke (yes/no), determined based on self-reported information. These factors were incorporated to assess their potential influence on the study’s outcomes.

### Statistical analyses

Data acquisition and calculations were carried out using R 4.2.1 software, while all analyses were performed using SPSS 26.0 software. To account for the complex survey design of NHANES data, we adhered to survey guidelines and applied appropriate sampling weights. Participants were categorized into two groups based on their HP antibody status.

In our study, we utilized “mean ± standard deviation” (mean ± SD) to represent quantitative data with a normal distribution. To test for differences between groups, we employed one-way analysis of variance (F). For quantitative data that did not follow a normal distribution, we expressed them as “median and quartiles” [M (Q1, Q3)] and compared groups using the Mann-Whitney U test. Categorical variables were described as “n (%)”, and group comparisons were conducted using the chi-square test (χ2). Statistical significance was considered at p-values less than 0.05.

Each dietary factor was classified into one of four categories based on quartiles of the score (P_25_, P_50_, P_75_), which were represented by numbers 1 to 4 (1 = < P_25_、2 = P_25_ ∼ P_50_、3 = P_50_ ∼ P_75_、4 = ≥ P_75_ percentile). Higher scores indicated higher intake of that dietary factor. Differences in the characteristics of each dietary pattern were assessed using one-way ANOVA for continuous variables and the chi-square test (χ2) for categorical variables. Multivariate logistic regression was employed to examine the relationship between dietary patterns and HP infection status. The independent variables were the quartiles of the factor scores for the identified dietary patterns, with the first quartile serving as the reference for each dietary pattern. Additionally, we investigated the association between specific food groups (Solid Fats, Refined Grains, Cheese, Added Sugars) and HP infection status.

## Results

### Characteristics of the study sample by HP infection status

A total of 2,952 individuals participated in our study, and Table 1 presents the baseline data for all participants categorized by their HP infection status. The median age of the participants was 51.0 years (with a range of 37.0 to 66.0), and the overall HP positivity rate was 44.9%. Individuals with HP infection tended to be female (*P* < 0.001), older (*P* < 0.001), have a higher BMI (*P* < 0.001), possess lower education levels (*P* < 0.001), have lower income (*P* < 0.001), smoke (*P* < 0.001), consume alcohol more frequently (*P* < 0.001), and had a higher prevalence of hypertension, diabetes, heart disease, and stroke (*P* < 0.001).

**Table 1:** Baseline characteristics of study population between HP seropositive group and negative group ^1^

*n*	Total	HP-	HP+	*p*
	2952	1627	1325	
**Age(year)** ^**2**^	51 (37,66)	47 (34,64)	55 (41,68)	< 0.001
**Gender,%**				< 0.001
Female	1475(51.3)	827(50.8)	648(52.3)	
Male	1477(48.7)	800(49.2)	677(47.7)	
**Race,%**				< 0.001
MA(Mexican American)	769(5.7)	248(3.2)	521(11.4)	
NHB(Non-Hispanic Black)	507(9.0)	214(5.9)	293(16.1)	
NHW(Non-Hispanic White)	1409(73.7)	1049(83.3)	360(51.9)	
OH(Other Hispanic)	183(7.1)	75(4.5)	708(13.1)	
OR(Other Race - Including Multi-Racial)	84(4.4)	41(3.1)	43(7.6)	
**Education level, %**				< 0.001
Less Than 9th Grade	524(6.0)	121(14.9)	403(2.2)	
9-11th Grade	555(15.7)	235(12.3)	320(23.2)	
High School Grad/GED or Equivalent	665(26.3)	409(26.0)	256(27.0)	
Some College or AA degree	697(28.7)	471(31.9)	226(21.5)	
College Graduate or above	511(23.3)	391(277.6)	120(13.4)	
**BMI,%**				< 0.001
<18.5	38(2.2)	15(1.8)	23(2.4)	
18.5–25	866(32.5)	348(31.5)	518(33.0)	
25–30	1076(35.2)	513(35.7)	563(34.9)	
≥ 30	972(30.1)	449(31.0)	523(29.7)	
**PIR,%**				< 0.001
<1.3	853(21.9)	341(17.1)	512(32.8)	
1.3–3.5	1138(42.6)	604(49.2)	534(27.8)	
>3.5	961(35.5)	682(33.7)	279(39.4)	
**Smoke,%**				< 0.001
Never	1519(50.3)	866(52.9)	653(44.4)	
Former	816(24.7)	445(24.5)	371(25.1)	
now	617(25.0)	316(22.6)	301(30.4)	
**Alcohol user,%**				< 0.001
Never	427(11.7)	213(10.6)	214(14.3)	
former	616(16.2)	297(13.9)	319(21.6)	
mild	966(35.0)	588(37.6)	378(29.0)	
moderate	405(16.8)	249(17.4)	156(15.5)	
heavy	538(20.3)	280(20.6)	258(19.7)	
**Hypertension,%**				< 0.001
No	1670(66.1)	980(68.2)	690(61.4)	
Yes	1282(33.9)	647(31.8)	635(38.6)	
**DM,%**				< 0.001
DM	395(8.5)	164(7.0)	231(12.1)	
IFG	118(3.0)	60(2.8)	58(3.4)	
No	2439(88.5)	1403(90.3)	1036(84.5)	
**Heart attack,%**				< 0.001
No	2820(96.0)	1563(96.4)	1257(95.1)	
Yes	132(4.0)	64(3.6)	68(4.9)	
**Stroke,%**				< 0.001
No	2853(97.8)	1583(98.2)	1270(97.0)	
Yes	99(2.2)	44(1.8)	55(3.0)	

^1^All results were survey-weighted except for counts of categorical variables ^2^Median (25%,75%) PIR, Poverty income ratio; MA, Mexican American; OH, Other Hispanic; NHW, Non-Hispanic White; NHB, Non-Hispanic Black; OR, Other race; DM, Diabetes Mellitus; IFG, Impaired Fasting Glucose

### Description of dietary patterns

Following varimax rotation, factor analysis identified three dietary patterns, and the primary food factor loadings for each pattern are presented in Table 2. These three dietary patterns collectively explained 22.1% of the total variance. Based on their contributions to the total variance, the three dietary patterns were defined as follows: Factor 1 was labeled the “High fats and high sugars pattern,” characterized by dietary intake primarily consisting of solid fats, refined grains, cheese, and added sugars. Factor 2 was identified as the “Vegetarian pattern,” characterized by a dietary intake predominantly composed of fruits, fruit juices, and whole grains. Factor 3 was designated the “Healthy pattern,” marked by dietary intake primarily featuring dark vegetables, other vegetables, nuts, and vegetable oils.

**Table 2:** Factor loadings for food intake patterns in the NHANES 1999–2000

Food groupings	F1: High fats and high sugars pattern	F2: Vegetarian pattern	F3: Healthy pattern
Solid Fats	**0.795**	**-0.211**	-0.007
Refined Grains	**0.723**	0.085	-0.051
Cheese	**0.620**	-0.026	0.041
Added Sugars	**0.451**	**-0.271**	-0.033
Tomatoes and Tomato Products	**0.408**	0.103	0.149
Cured Meat (frankfurters, sausage, corned beef, cured ham and luncheon meat made from beef, pork, poultry)	**0.357**	-0.111	-0.054
Milk	**0.341**	0.185	-0.090
Oils	**0.272**	-0.058	**0.755**
Other Vegetables	0.042	**0.212**	**0.569**
Nuts and Seeds	0.130	0.033	**0.522**
Dark Green Vegetables	-0.072	**0.266**	**0.432**
Other Red and Orange Vegetables (excludes, tomatoes)	-0.086	0.093	**0.366**
Eggs	0.153	-0.083	-0.003
Organ Meat (from beef, veal, pork, lamb, game, poultry)	0.018	-0.040	-0.021
Friut	0.017	**0.704**	0.039
Fruit Juice	0.016	**0.610**	0.010
Whole Grains	-0.023	**0.454**	0.050
Potatoes (white potatoes)	0.027	**-0.409**	**0.242**
Red Meat (beef, veal, pork, lamb, game)	0.129	**-0.335**	0.150
Soy Products	-0.020	**0.250**	0.140
Legumes	0.186	**0.211**	-0.099
Yogurt	0.014	0.116	0.049
Alcoholic Drinks	-0.002	-0.140	0.163
Seafood Low in n-3 Fatty Acids	-0.061	-0.054	0.162
Seafood High in n-3 Fatty Acids	-0.067	-0.030	0.162
Poultry (chicken, turkey, other fowl)	-0.096	0.021	0.114
Other Starchy Vegetables (excludes white potatoes)	-0.179	-0.091	0.094
Eigenvalue	2.384	1.826	1.744
Variance explained (%)	8.832	6.765	6.457
Cumulative variance explained (%)	8.832	15.597	22.055

*Note* Factor loadings > 0.2 or <-0.2 are in bold type

### Characteristics of the study sample by dietary patterns

Dietary pattern scores were categorized into quartiles to facilitate comparisons across different intake levels. The three dietary patterns exhibit their fundamental characteristics across these quartiles.

Individuals in the highest quartile of the “High fats and high sugars pattern” were generally younger, more likely to be male, more educated, had a higher BMI, higher income, were more frequent smokers and drinkers, and had a higher prevalence of high blood pressure, heart disease, and stroke, but a lower prevalence of diabetes (Supplementary Table 1).Participants with the highest scores in the “Vegetarian pattern” were older, more likely to be female, more educated, had a lower BMI, higher income, were less frequent smokers and drinkers, and had a higher prevalence of hypertension, diabetes, and heart disease, but a lower likelihood of stroke (Supplementary Table 2).Individuals with the highest scores in the “Healthy pattern” were younger, more likely to be male, more educated, had a lower BMI, higher income, smoked and drank less, and had a lower prevalence of hypertension, diabetes, heart disease, and stroke (Supplementary Table 3).

### Association between dietary patterns and HP infection status

The associations between dietary patterns and HP infection status are presented in Table 3. The “High fats and high sugars pattern” exhibited a negative association with the overall prevalence of HP infection (*P* < 0.001). After adjusting for confounding factors, it was observed that the prevalence of HP infection among participants in the second quartile of the “High fats and high sugars pattern” was 1.18 times higher than that of participants in the first percentile (95% CI, 1.177–1.179). However, as the score increased, the “High fats and high sugars pattern” gradually transitioned into a protective factor, with a multivariable-adjusted odds ratio (OR) for the prevalence of HP infection in participants in the highest quartile being 0.689 (95% CI, 0.688–0.690).

The “Vegetarian pattern” and the “Healthy pattern” were both linked to a reduced prevalence of HP infection (*P* < 0.001), and these associations remained consistent after adjusting for confounding variables, with ORs of 0.802 (95% CI, 0.801–0.803) and 0.717 (95% CI, 0.716–0.718), respectively.

**Table 3:** The association between dietary pattern score quartile and HP infection status ^1^

	Quartile of Dietary Pattern Scores^2^				*p* for Trend
	Q1	Q2	Q3	Q4	
High fats and high sugars pattern					
Model 1	1.0(Ref.)	1.015(1.014,1.016)	0.809(0.808,0.810)	0.520(0.519,0.520)	< 0.001
Model 2	1.0(Ref.)	1.178(1.177,1.179)	0.995(0.994,0.996)	0.689(0.688,0.690)	< 0.001
**Vegetarian pattern**					
Model 1	1.0(Ref.)	0.855(0.854,0.856)	0.677(0.676,0.678)	0.804(0.803,0.805)	< 0.001
Model 2	1.0(Ref.)	0.837(0.836,0.838)	0.618(0.617,0.619)	0.802(0.801,0.803)	< 0.001
**Healthy pattern**					
Model 1	1.0(Ref.)	0.773(0.772,0.774)	0.602(0.601,0.603)	0.481(0.480,0.482)	< 0.001
Model 2	1.0(Ref.)	0.899(0.898,0.900)	0.733(0.732,0.733)	0.717(0.716,0.718)	< 0.001

^1^ All results were survey-weighted except for sample counts ^2^ The independent variables were the quartiles of the factor scores for the identified dietary patterns, using the first quartile as the reference for each dietary pattern CI, confidence interval; Q, quartiles. Model 1: unadjusted; Model 2: adjusted by age, gender, race, education level, BMI, poverty income ratio, smoke, alcohol user, hypertension, diabetes mellitus, heart attack, stroke

### Association between solid fats, refined grains, cheese, added sugars and HP infection status

As solid fats, refined grains, cheese, and added sugars are fundamental components of the “High fats and high sugars pattern,” we examined their intake in relation to HP infection status(Table 4). Adjusted refined grains and added sugars consistently demonstrated as risk factors for HP infection (*P* < 0.001). In contrast, solid fats and cheese from animal sources exhibited a protective effect with increasing intake. The ORs decreased from 1.189 (95% CI, 1.187–1.190) to 0.717 (95% CI, 0.716–0.718) for solid fats and from 1.116 (95% CI, 1.115–1.118) to 0.863 (95% CI, 0.862–0.864) for cheese, respectively.

**Table 4:** Associations between the specific food group scores and HP infection status^1,2^

	Quartile of Dietary Pattern Scores^3^				*p* for Trend
	Q1	Q2	Q3	Q4	
Solid Fats					
Model 1	1.0(Ref.)	1.183(1.182,1.184)	0.860(0.859,0.861)	0.822(0.821,0.823)	< 0.001
Model 2	1.0(Ref.)	1.174(1.173,1.176)	0.844(0.843,0.845)	0.778(0.776,0.779)	< 0.001
Model 3	1.0(Ref.)	1.189(1.187,1.190)	0.848(0.847,0.849)	0.717(0.716,0.718)	< 0.001
**Refined Grains**					
Model 1	1.0(Ref.)	1.119(1.118,1.120)	1.167(1.166,1.168)	0.956(0.955,0.957)	< 0.001
Model 2	1.0(Ref.)	1.150(1.149,1.152)	1.217(1.216,1.218)	1.030(1.029,1.031)	< 0.001
Model 3	1.0(Ref.)	1.197(1.195,1.198)	1.336(1.334,1.337)	1.045(1.044,1.046)	< 0.001
**Cheese**					
Model 1	1.0(Ref.)	0.819(0.818,0.820)	0.792(0.791,0.792)	0.542(0.542,0.543)	< 0.001
Model 2	1.0(Ref.)	0.862(0.861,0.863)	0.776(0.775,0.777)	0.553(0.553,0.554)	< 0.001
Model 3	1.0(Ref.)	1.116(1.115,1.118)	1.036(1.034,1.037)	0.863(0.862,0.864)	< 0.001
**Added Sugars**					
Model 1	1.0(Ref.)	0.985(0.984,0.986)	0.889(0.888,0.890)	0.893(0.892,0.894)	< 0.001
Model 2	1.0(Ref.)	0.977(0.976,0.978)	0.827(0.826,0.828)	0.785(0.784,0.786)	< 0.001
Model 3	1.0(Ref.)	1.088(1.087,1.089)	1.099(1.098,1.100)	1.014(1.013,1.015)	< 0.001

^1^ All results were survey-weighted except for sample counts ^2^ Selected covariates include consumption of solid fats, refined grains, cheese, and added sugars from the high fat and high sugar patterns ^3^ The dependent variable is the determined quartile of the factor scores for the selected food groups, using the first quartile as the reference for each food group CI, confidence interval; Q, quartiles. Model 1: unadjusted; Model 2: adjusted by vegetarian pattern, healthy pattern; Model 3: adjusted by vegetarian pattern, healthy pattern, age, gender, race, education level, BMI, poverty income ratio, smoke, alcohol user, hypertension, diabetes mellitus, heart attack, stroke

## Discussion

A total of 2,952 participants who met the criteria were included in this cross-sectional study. This study represents the first attempt to assess the association between dietary patterns and HP infection status in U.S. adults. Our findings indicated that the “Vegetarian pattern” and the “Healthy pattern” were negatively associated with HP infection rates. Conversely, the “High fats and high sugars pattern,” characterized by solid fats, refined grains, cheese, and added sugars, transitioned from a risk factor to a protective factor for HP infection as intake increased. Further analyses indicated that within the “High fats and high sugars pattern,” refined grains and added sugars were the primary factors contributing to an increased risk of HP infection. On the other hand, solid fats and cheese from animal sources exhibited potential protective effects.

The protective effect observed in the “High fats and high sugars pattern” may not be entirely consistent with previous research. However, this discrepancy can be explained by our further analysis of food groups. Our findings suggest that solid fats and cheeses from animal sources ultimately play a protective role. Foods of animal source are rich in vitamins and trace minerals such as vitamin A, D, selenium and zinc, which inhibit the colonization of HP and the inflammation induced by HP [21], and even influence HP eradication [22]. In a recent case-control study in Iran [23], there was no significant difference in fat intake between healthy participants and HP-infected individuals when dietary intake was used as a variable (*P* = 0.398). However, when the variable in this study was the food group, animal foods (including meat and processed meats) were considered risk factors for HP infection. We believe that broadly grouping meat with processed meats such as sausages may obscure the benefits of animal-based foods. As another Iranian study concluded by grouping meat samples more carefully [24], it is sausages, burgers, and fatty mayonnaise that are truly positively associated with HP infection, rather than meat and fat. The effects of refined carbohydrates and added sugars are similar to those of processed meats, as these ultra-processed foods are often overloaded with salt and sugar for taste, preservation, and cost, which tend to increase the colonization of HP as well as interfere with the protective effects of intragastric mucus and gastric mucosa [13, 25]. Animal foods have been shown to provide better nutritional status to combat HP infection [26, 27], and recent research in other areas has shifted the previous view of animal foods [28, 29]. However, it is undeniable that consuming too much animal food can bring about obesity and a variety of chronic metabolic diseases, so our results need to be taken with a grain of salt, besides more research is needed to elucidate its role.

The vegetarian pattern, characterized by a high intake of fruits and vegetables, typically acts as a protective factor against various diseases, thus diminishing the role of HP [30, 31]. This protective effect is attributed to the presence of antioxidants like carotenoids, vitamin C, and vitamin E in fruits and vegetables, which also help deter the progression of HP-related diseases such as atrophic gastritis [32]. Nevertheless, it’s important to acknowledge certain shortcomings in vegan diets. People following plant-based diets may experience insufficient intake of protein, vitamins B12 and D, calcium, iron, and zinc due to either their low levels in plant-based foods or limited absorption [33]. Deficiencies in these macronutrients, vitamins, and micronutrients not only increase the risk of HP infection but also elevate the risk of conditions like hemorrhagic stroke and fractures [34]. The efficiency of protein utilization tends to decrease with age, and abstaining from animal foods may not fulfill the nutritional needs of older adults [35]. More significantly, these nutrient deficiencies are often more pronounced in children and pregnant or lactating women, leading to serious consequences like megaloblastic anemia and irreversible neurological damage [36].

The quality of a vegan diet plays a crucial role in reducing the risk of various chronic diseases. In the absence of dietary and medical supervision, the consumption of highly processed plant-based products can compromise the effectiveness of a plant-based diet and even have adverse effects. Additionally, this dietary pattern often includes the consumption of raw vegetables and fruits. As a foodborne [37] and waterborne [38] pathogen, HP can be transmitted to humans through this pathway. HP has the capacity to form biofilms and thrive as colonies on the surfaces of vegetables, greatly extending its survival [39]. This is why frequent consumption of raw vegetables like cucumbers [40], tomatoes, and peppers [41] has been associated with an increased risk of HP infection in some studies. However, relatively speaking, groups with higher fruit and vegetable consumption are usually more affluent and better educated [42], especially since our study population came from a developed country like the United States, which typically boasts better sanitary conditions compared to many other countries. The protective effect ultimately results from the combined influence of dietary and socio-economic factors.

We also note that some researchers have questioned the relationship between HP and diet [43], suggesting that HP is usually acquired in childhood and that dietary factors may play a role later in life after HP infection. The prevalence of HP infection in children has been reported to about 33% (with variations in different regions) [44], and the difference in the prevalence with adults suggests that HP infection status in adulthood may arise from two sources, maintenance of infection in childhood and acquisition in adulthood. There is still no consensus on screening in childhood [45], and obtaining and following up this part of the data becomes difficult. Children’s dietary habits change with age, in addition to the influence of geographic factors, economic status, and level of education, so the current study could not clarify the difference between the role of diet in the maintenance and acquisition of HP infection. However, diet remains worthy of investigation as the cheapest and most easily achievable variable factor in HP infection prevention and treatment for adults with stabilized dietary habits and physical status.

In summary, our research is the first to investigate the relationship between dietary patterns and the risk of HP infection among U.S. adults. Previous studies conducted in the provincial capital of Zhejiang, China [16] were limited by differences in economic income and education levels compared to other provinces and cities, which may affect the generalizability of the results. However, NHANES provided a representative sample of Americans through its sampling methodology, allowing our findings to be more widely applicable. Notably, in another study on dietary patterns [15], no significant association was observed between any food group and *Helicobacter pylori* infection, whereas our study identified distinct roles of specific food groups in HP infection. Nonetheless, our study has some limitations. Firstly, it was cross-sectional, and as such, it cannot establish a causal relationship between dietary patterns and the risk of HP infection. Secondly, there may be recall bias in dietary assessment due to the use of the Food Frequency Questionnaire (FFQ), which could lead to inaccuracies in food categorization and intake estimation. Thirdly, HP infection status was determined solely by HP-specific IgG antibodies, and this method may introduce bias in the diagnosis of infection.

## Conclusion

Our study revealed that dietary patterns play a significant role in the prevalence of HP infection among U.S. adults. Specifically, a Vegetarian dietary pattern characterized by high fruit, fruit juice, and whole grain consumption, as well as a healthy dietary pattern rich in vegetables, nuts, and vegetable oils, were associated with a reduced risk of HP infection status. Interestingly, a dietary pattern high in fats and sugars, featuring increased intake of solid fats, refined grains, cheese, and added sugars, was also found to be a protective factor against HP infection. However, further analysis suggested that reducing the consumption of added sugars and refined grains, while not overly restricting the intake of cheese and solid fats, may help lower the risk of HP infection status.

### Electronic supplementary material

Below is the link to the electronic supplementary material.

Supplementary Material 1

## Data Availability

All data of the study are presented in the text or supplementary materials. The datasets analyzed during the current study were publicly available from the NAHNES. Data from the NHANES can be found at https://www.cdc.gov/nchs/nhanes/index.htm. The FPED is available at https://www.ars.usda.gov/northeast-area/beltsville-md-bhnrc/beltsville-human-nutrition-research-center/food-surveys-research-group/docs/fped-overview/.

## Abbreviations

NHANES: National Health and Nutrition Examination Survey
HP: *Helicobacter pylori*
PIR: Poverty Income Ratio
MA: Mexican American
OH: Other Hispanic
NHW: Non-Hispanic White
NHB: Non-Hispanic Black
OR: Other race
DM: Diabetes Mellitus
IFG: Impaired Fasting Glucose
Q: Quartiles
CI: Confidence interval
