# Supplementary material for: The intake of solid fat and cheese may be associated with a reduced risk of Helicobacter pylori infection status: a cross-sectional study based on NHANES 1999–2000
Source: BMC Infect Dis. 2024 May 14;24:493. doi: 10.1186/s12879-024-09392-z (PMC11092062; doi:10.1186/s12879-024-09392-z)
Supplement: Supplementary file 1 — Supplementary Material 1 [file 12879_2024_9392_MOESM1_ESM.docx]

| **Supplementary Table 1.** Characteristics of study participants by quartiles of dietary pattern scores in “high fats, refined grains, and high sugars” pattern^1^ | | | | | |
| --- | --- | --- | --- | --- | --- |
| Characteristics | High fats and high sugars pattern | | | | p value |
|  | Q1 | Q2 | Q3 | Q4 |  |
| **Age(year)^2^** | 60 (42,70) | 53 (39,68) | 48 (34,64) | 42 (32,54) | <0.001 |
| **Gender,%** |  |  |  |  | <0.001 |
| Female | 522(65.0) | 436(62.9) | 322(44.7) | 195(32.6) |  |
| Male | 321(35.0) | 332(37.1) | 411(55.3) | 413(67.4) |  |
| **Race,%** |  |  |  |  | <0.001 |
| MA(Mexican American) | 225(5.2) | 199(6.2) | 209(6.5) | 136(4.9) |  |
| NHB(Non-Hispanic Black) | 193(12.6) | 126(8.1) | 101(7.9) | 87(7.5) |  |
| NHW(Non-Hispanic White) | 331(66.7) | 379(74.5) | 370(76.4) | 329(77.2) |  |
| OH(Other Hispanic) | 59(7.9) | 39(6.4) | 40(6.4) | 45(7.7) |  |
| OR(Other Race - Including Multi-Racial) | 35(7.6) | 25(4.8) | 13(2.7) | 11(2.7) |  |
| **Education level, %** |  |  |  |  | <0.001 |
| Less Than 9th Grade | 209(9.7) | 128(5.8) | 121(5.6) | 66(3.1) |  |
| 9-11th Grade | 172(15.8) | 148(17.2) | 127(15.9) | 108(13.7) |  |
| High School Grad/GED or Equivalent | 176(27.2) | 189(26.7) | 165(24.0) | 135(27.2) |  |
| Some College or AA degree | 156(25.7) | 188(30.0) | 184(28.6) | 169(30.5) |  |
| College Graduate or above | 130(21.5) | 115(20.3) | 136(25.9) | 130(25.5) |  |
| **BMI,%** |  |  |  |  | <0.001 |
| ＜18.5 | 14(2.4) | 9(2.7) | 7(1.2) | 8(2.6) |  |
| 18.5-25 | 243(33.3) | 219(31.3) | 224(34.7) | 180(30.8) |  |
| 25-30 | 313(35.6) | 287(36.8) | 257(32.6) | 219(35.6) |  |
| ≥30 | 273(28.6) | 253(29.2) | 245(31.5) | 201(31.0) |  |
| **PIR,%** |  |  |  |  | <0.001 |
| ＜1.3 | 285(24.4) | 220(23.5) | 202(22.4) | 146(17.2) |  |
| 1.3-3.5 | 306(33.8) | 301(35.5) | 292(35.1) | 239(37.5) |  |
| ＞3.5 | 252(41.8) | 247(41.0) | 239(42.4) | 223(45.3) |  |
| **Smoke,%** |  |  |  |  | <0.001 |
| Never | 461(53.5) | 384(49.8) | 380(49.4) | 294(48.6) |  |
| Former | 220(21.5) | 226(26.4) | 206(27.7) | 164(23.2) |  |
| now | 162(25.0) | 158(23.8) | 147(23.0) | 150(28.1) |  |
| **Alcohol user,%** |  |  |  |  | <0.001 |
| Never | 156(12.3) | 121(13.9) | 95(11.6) | 55(9.0) |  |
| former | 202(18.1) | 150(14.2) | 152(16.4) | 112(16.2) |  |
| mild | 250(35.2) | 250(34.0) | 261(36.9) | 205(33.6) |  |
| moderate | 95(14.7) | 112(18.0) | 95(15.0) | 103(19.4) |  |
| heavy | 140(19.6) | 135(19.8) | 130(20.0) | 133(21.8) |  |
| **Hypertension,%** |  |  |  |  | <0.001 |
| No | 407(62.8) | 421(63.6) | 420(64.2) | 422(73.9) |  |
| Yes | 436(37.2) | 347(36.4) | 313(35.8) | 186(26.1) |  |
| **DM,%** |  |  |  |  | <0.001 |
| DM | 156(12.5) | 108(8.2) | 74(7.5) | 57(5.8) |  |
| IFG | 26(2.3) | 42(4.0) | 29(2.8) | 21(2.7) |  |
| No | 661(85.2) | 618(87.8) | 630(89.6) | 530(91.5) |  |
| **Heart attack,%** |  |  |  |  | <0.001 |
| No | 791(95.0) | 735(96.0) | 700(95.4) | 594(97.6) |  |
| Yes | 52(5.0) | 33(4.0) | 33(4.6) | 14(2.4) |  |
| **Stroke,%** |  |  |  |  | <0.001 |
| No | 799(97.5) | 742(97.3) | 718(98.5) | 594(97.9) |  |
| Yes | 44(2.5) | 26(2.7) | 15(1.5) | 14(2.1) |  |
| ^1^All results were survey-weighted except for counts of categorical variables; | | | | |  |
| ^2^Median(25%,75%) |  |  |  |  |  |
| PIR, Poverty income ratio; MA, Mexican American; OH, Other Hispanic; NHW, Non-Hispanic White; NHB, Non-Hispanic Black; OR, Other race; DM, Diabetes Mellitus; IFG, Impaired Fasting Glucose; Q, quartiles | | | | | |

| **Supplementary Table 2.** Characteristics of study participants by quartiles of dietary pattern scores in “vegetarian pattern” pattern ^1^ | | | | | |
| --- | --- | --- | --- | --- | --- |
| Characteristics | Vegetarian pattern | | | | p value |
|  | Q1 | Q2 | Q3 | Q4 |  |
| **Age(year)^2^** | 42 (32.0,56.3) | 47 (33.5,63.0) | 54 (40.0,67.0) | 60 (42.0,71.0) | <0.001 |
| **Gender,%** |  |  |  |  | <0.001 |
| Female | 219(39.3) | 386(55.0) | 445(56.9) | 425(54.0) |  |
| Male | 373(60.7) | 357(45.0) | 349(43.1) | 398(46.0) |  |
| **Race,%** |  |  |  |  | <0.001 |
| MA(Mexican American) | 117(4.4) | 212(6.9) | 229(5.8) | 211(5.5) |  |
| NHB(Non-Hispanic Black) | 116(8.8) | 142(10.8) | 129(8.7) | 120(7.8) |  |
| NHW(Non-Hispanic White) | 309(76.1) | 326(69.6) | 364(73.9) | 410(75.2) |  |
| OH(Other Hispanic) | 30(5.0) | 42(7.3) | 58(9.1) | 53(7.0) |  |
| OR(Other Race - Including Multi-Racial) | 20(5.6) | 21(5.3) | 14(2.4) | 29(4.4) |  |
| **Education level, %** |  |  |  |  | <0.001 |
| Less Than 9th Grade | 75(5.1) | 120(5.9) | 165(6.6) | 164(6.5) |  |
| 9-11th Grade | 146(21.3) | 164(19.6) | 134(12.5) | 111(9.2) |  |
| High School Grad/GED or Equivalent | 162(32.3) | 181(27.6) | 163(23.9) | 159(21.4) |  |
| Some College or AA degree | 143(28.1) | 180(28.8) | 195(30.9) | 179(27.1) |  |
| College Graduate or above | 66(13.2) | 98(18.1) | 137(26.1) | 210(35.8) |  |
| **BMI,%** |  |  |  |  | <0.001 |
| ＜18.5 | 9(1.7) | 7(1.3) | 9(1.7) | 13(4.2) |  |
| 18.5-25 | 168(32.1) | 206(31.8) | 218(31.9) | 274(34.3) |  |
| 25-30 | 193(31.6) | 258(34.8) | 318(37.2) | 307(36.9) |  |
| ≥30 | 222(34.6) | 272(32.0) | 249(29.2) | 229(24.6) |  |
| **PIR,%** |  |  |  |  | <0.001 |
| ＜1.3 | 178(23.5) | 238(26.6) | 225(17.9) | 212(19.5) |  |
| 1.3-3.5 | 230(36.9) | 292(36.2) | 309(36.5) | 307(32.3) |  |
| ＞3.5 | 184(39.6) | 213(37.2) | 260(45.5) | 304(48.2) |  |
| **Smoke,%** |  |  |  |  | <0.001 |
| Never | 233(41.1) | 360(46.1) | 441(55.0) | 485(59.2) |  |
| Former | 133(17.0) | 205(25.5) | 230(27.3) | 248(29.1) |  |
| now | 226(41.9) | 178(28.4) | 123(17.7) | 90(11.7) |  |
| **Alcohol user,%** |  |  |  |  | <0.001 |
| Never | 56(7.4) | 107(12.9) | 132(12.9) | 132(13.7) |  |
| former | 100(14.2) | 158(16.6) | 186(18.5) | 172(15.5) |  |
| mild | 144(23.1) | 216(29.0) | 284(41.5) | 322(46.2) |  |
| moderate | 107(21.8) | 100(16.7) | 102(14.8) | 96(13.9) |  |
| heavy | 185(33.5) | 162(24.8) | 90(12.3) | 101(10.6) |  |
| **Hypertension,%** |  |  |  |  | <0.001 |
| No | 376(69.8) | 423(66.6) | 452(67.5) | 419(60.6) |  |
| Yes | 216(30.2) | 320(33.4) | 342(32.5) | 404(39.4) |  |
| **DM,%** |  |  |  |  | <0.001 |
| DM | 56(6.5) | 82(6.6) | 130(11.0) | 127(10.1) |  |
| IFG | 25(2.6) | 37(3.7) | 25(1.9) | 31(3.6) |  |
| No | 511(90.9) | 624(89.8) | 639(87.2) | 665(86.3) |  |
| **Heart attack,%** |  |  |  |  | <0.001 |
| No | 571(97.3) | 713(96.5) | 761(95.8) | 775(94.4) |  |
| Yes | 21(2.7) | 30(3.5) | 33(4.2) | 48(5.6) |  |
| **Stroke,%** |  |  |  |  | <0.001 |
| No | 583(99.0) | 722(98.5) | 751(95.8) | 797(97.9) |  |
| Yes | 9(1.0) | 21(1.5) | 43(4.2) | 26(2.1) |  |
| ^1^All results were survey-weighted except for counts of categorical variables; | | | | |  |
| ^2^Median(25%,75%) |  |  |  |  |  |
| PIR, Poverty income ratio; MA, Mexican American; OH, Other Hispanic; NHW, Non-Hispanic White; NHB, Non-Hispanic Black; OR, Other race; DM, Diabetes Mellitus; IFG, Impaired Fasting Glucose; Q, quartiles | | | | | |

| **Supplementary Table 3.** Characteristics of study participants by quartiles of dietary pattern scores in “healthy pattern” pattern^1^ | | | | | |
| --- | --- | --- | --- | --- | --- |
| Characteristics | Healthy pattern | | | | p value |
|  | Q1 | Q2 | Q3 | Q4 |  |
| **Age(year)^2^** | 51 (34.8,67.0) | 52 (36.0,68.0) | 51 (38.0,66.0) | 49 (37.0,63.0) | <0.001 |
| **Gender,%** |  |  |  |  | <0.001 |
| Female | 440(55.5) | 425(57.4) | 355(49.1) | 255(43.3) |  |
| Male | 408(44.5) | 347(42.6) | 372(50.9) | 350(56.7) |  |
| **Race,%** |  |  |  |  | <0.001 |
| MA(Mexican American) | 293(8.1) | 205(6.3) | 158(4.5) | 113(3.8) |  |
| NHB(Non-Hispanic Black) | 153(11.5) | 148(10.3) | 128(9.2) | 78(5.2) |  |
| NHW(Non-Hispanic White) | 312(65.7) | 352(71.6) | 372(74.4) | 373(83.1) |  |
| OH(Other Hispanic) | 72(10.9) | 43(7.3) | 41(6.4) | 27(3.8) |  |
| OR(Other Race - Including Multi-Racial) | 18(3.7) | 24(4.5) | 28(5.5) | 14(4.1) |  |
| **Education level, %** |  |  |  |  | <0.001 |
| Less Than 9th Grade | 228(9.8) | 147(7.0) | 97(4.7) | 52(2.6) |  |
| 9-11th Grade | 192(20.8) | 140(15.7) | 129(14.3) | 94(11.8) |  |
| High School Grad/GED or Equivalent | 167(26.4) | 185(27.3) | 188(31.2) | 125(20.3) |  |
| Some College or AA degree | 178(27.9) | 174(28.9) | 171(25.9) | 174(32.2) |  |
| College Graduate or above | 83(15.1) | 126(21.0) | 142(23.8) | 160(33.2) |  |
| **BMI,%** |  |  |  |  | <0.001 |
| ＜18.5 | 12(3.5) | 9(1.8) | 8(1.6) | 9(1.9) |  |
| 18.5-25 | 237(30.6) | 222(32.4) | 214(32.1) | 193(35.1) |  |
| 25-30 | 323(35.0) | 282(36.1) | 252(33.8) | 219(35.8) |  |
| ≥30 | 276(30.9) | 259(29.7) | 253(32.5) | 184(27.2) |  |
| **PIR,%** |  |  |  |  | <0.001 |
| ＜1.3 | 313(30.6) | 244(25.0) | 184(19.1) | 112(12.9) |  |
| 1.3-3.5 | 345(39.1) | 298(36.0) | 269(33.2) | 226(33.6) |  |
| ＞3.5 | 190(30.4) | 230(39.0) | 274(47.7) | 267(53.5) |  |
| **Smoke,%** |  |  |  |  | <0.001 |
| Never | 444(50.0) | 427(53.5) | 352(47.4) | 296(50.5) |  |
| Former | 226(22.6) | 180(21.4) | 211(25.2) | 199(29.6) |  |
| now | 178(27.4) | 165(25.1) | 164(27.4) | 110(19.9) |  |
| **Alcohol user,%** |  |  |  |  | <0.001 |
| Never | 144(14.9) | 116(13.1) | 108(10.8) | 59(8.1) |  |
| former | 200(18.3) | 178(17.7) | 142(16.9) | 96(12.0) |  |
| mild | 246(31.6) | 246(34.3) | 233(32.9) | 241(41.0) |  |
| moderate | 98(12.8) | 105(18.3) | 108(17.1) | 94(18.9) |  |
| heavy | 160(22.3) | 127(16.6) | 136(22.4) | 115(20.0) |  |
| **Hypertension,%** |  |  |  |  | <0.001 |
| No | 500(69.0) | 423(65.5) | 395(63.4) | 352(66.7) |  |
| Yes | 348(31.0) | 349(34.5) | 332(36.6) | 253(33.3) |  |
| **DM,%** |  |  |  |  | <0.001 |
| DM | 110(8.4) | 107(8.8) | 107(10.2) | 71(6.6) |  |
| IFG | 30(2.7) | 35(3.1) | 30(2.7) | 23(3.4) |  |
| No | 708(88.9) | 630(88.0) | 590(87.2) | 511(90.0) |  |
| **Heart attack,%** |  |  |  |  | <0.001 |
| No | 809(95.7) | 741(96.9) | 689(95.7) | 581(95.8) |  |
| Yes | 39(4.3) | 31(3.1) | 38(4.3) | 24(4.2) |  |
| **Stroke,%** |  |  |  |  | <0.001 |
| No | 814(97.4) | 740(97.4) | 707(98.0) | 592(98.4) |  |
| Yes | 34(2.6) | 32(2.6) | 20(2.0) | 13(1.6) |  |
| ^1^All results were survey-weighted except for counts of categorical variables; | | | | |  |
| ^2^Median(25%,75%) |  |  |  |  |  |
| PIR, Poverty income ratio; MA, Mexican American; OH, Other Hispanic; NHW, Non-Hispanic White; NHB, Non-Hispanic Black; OR, Other race; DM, Diabetes Mellitus; IFG, Impaired Fasting Glucose; Q, quartiles | | | | | |
